# NMR Reveals the Conformational Changes of Cytochrome C upon Interaction with Cardiolipin

**DOI:** 10.3390/life11101031

**Published:** 2021-09-30

**Authors:** Jianhua Zhan, Guangqing Zhang, Xin Chai, Qinjun Zhu, Peng Sun, Bin Jiang, Xin Zhou, Xu Zhang, Maili Liu

**Affiliations:** 1 Key Laboratory of Magnetic Resonance in Biological Systems, State Key Laboratory of Magnetic Resonance and Atomic and Molecular Physics, National Center for Magnetic Resonance in Wuhan, Wuhan Institute of Physics and Mathematics, Innovation Academy for Precision Measurement of Science and Technology, Chinese Academy of Sciences, Wuhan 430071, China; zhanjianhua17@mails.ucas.ac.cn (J.Z.); zhangguangqing15@mails.ucas.ac.cn (G.Z.); chaixin@apm.ac.cn (X.C.); zhuqinjun@apm.ac.cn (Q.Z.); jbin@wipm.ac.cn (B.J.); xinzhou@wipm.ac.cn (X.Z.); 2 University of Chinese Academy of Sciences, Beijing 100049, China; 3 Philips Healthcare, No. 1628, Zhongshan Road, Wuhan 430071, China; peng.sun@philips.com; 4 Wuhan National Laboratory for Optoelectronics, Huazhong University of Science and Technology, Wuhan 430071, China

**Keywords:** NMR, cytochrome c, cardiolipin, conformation, methyl

## Abstract

Conformational change of cytochrome c (cyt c) caused by interaction with cardiolipin (CL) is an important step during apoptosis, but the underlying mechanism is controversial. To comprehensively clarify the structural transformations of cyt c upon interaction with CL and avoid the unpredictable alias that might come from protein labeling or mutations, the conformation of purified yeast iso–1 cyt c with natural isotopic abundance in different contents of CL was measured by using NMR spectroscopy, in which the trimethylated group of the protein was used as a natural probe. The data demonstrate that cyt c has two partially unfolded conformations when interacted with CL: one with Fe–His33 coordination and the other with a penta–coordination heme. The Fe–His33 coordination conformation can be converted into a penta–coordination heme conformation in high content of CL. The structure of cyt c becomes partially unfolded with more exposed heme upon interaction with CL, suggesting that cyt c prefers a high peroxidase activity state in the mitochondria, which, in turn, makes CL easy to be oxidized, and causes the release of cyt c into the cytoplasm as a trigger in apoptosis.

## 1. Introduction

Cyt c is a vital multifunctional protein critical for both respiration and apoptosis [1]. During apoptosis, the interaction between cyt c and CL has been suggested to play a key role in the release of cyt c from the mitochondria [2]. CL is abundant in the inner mitochondrial membrane (IMM), accounting for 25% of the total lipid content [3]. It migrates from the IMM to the outer mitochondrial membrane (OMM) in the preapoptotic stage [4,5]. CL is negatively charged, while the surface of cyt c is positively charged. Cyt c interacts with CL through four binding sites, namely Site A (Lys72, Lys73, Lys86, and Lys87) [6,7,8], Site C (Asn52) [9], Site L (Lys22, Lys27, His26, and His33) [10], and Site N (Phe36, Gly37, Thr58, Trp59, and Lys60) [11].

The conformation of cyt c transforms into some conformers with extremely high peroxidase activity when it binds to CL [12], which, in turn, makes it possible to oxidize CL as a peroxidase and then alter the permeability of the OMM in response to the release of the protein [13]. The peroxidase activity of cyt c has always been accompanied by the conformational transition [14].

However, the conformational changes of cyt c is still a controversial issue. Based on hydrogen–deuterium exchange mass spectrometry and ion mobility–mass spectrometry results, cyt c has been found to adopt a more compact configuration, with no evidence of global unfolding when bound to CL [15]. However, results from time–resolved fluorescence resonance energy transfer experiments suggest that cyt c becomes partially unfolded when binding with CL, and the Fe–Met80 bond of cyt c is broken and replaced by other residues [12,16]. Some studies propose that Met80 is replaced by a lysine [15,17], while other studies suppose it is replaced by a histidine [18]. There are still a few studies that suggest the Fe–Met80 bond is merely broken and results in a penta–coordination heme [19].

This controversy may exist because the altered conformations of cyt c upon CL interaction are heterogeneously assembled, undergo exchange, and are sensitive to the conditions of the experiments [20]. Spectroscopies that are sensitive to specific conformations may result in partial loss of information. NMR spectroscopy can simultaneously identify and monitor different molecules or conformers “without prejudice” and provide protein structure information at atomic resolution. It has been successfully used to detect the binding site of cyt c upon interaction with CL [21,22]. However, it is difficult to be used to observe structural changes at a high CL/cyt c molar ratio, as cyt c, when bound to large, slow–tumbling liposomes, has a higher correlation time, causing a decrease in NMR signal intensity [23,24], which, in turn, results in the disappearance of ^1^H–^15^N HSQC signals of cyt c upon interaction with high content of CL [22]. Additionally, data obtained from mutations or labeled molecules may induce alias information, as it was found that the structure of the usually used mutation of trimethyllysine 72 to alanine lost the Met80 heme ligand, causing a fourfold increase in peroxidase activity relative to monomeric cyt c [25], and the mutation of trimethyllysine 72 to alanine enhanced the His79–heme–mediated dynamics of iso–1 cyt c [26].

Herein, to obtain comprehensive information about the conformers of cyt c upon interaction with CL, and avoid the unpredictable alias results that might come from protein labeling or mutations, ^1^H–^13^C NMR was used to investigate the conformational change of purified yeast iso–1 cyt c with natural isotopic abundance upon interaction with CL. In yeast cells, though the downstream pathways of cyt c are not yet clear [27], it has also been found that cyt c is released from the mitochondria into the cytoplasm when it undergoes apoptosis induced by acetic acid [28,29], H_2_O_2_ [30], or electric fields [31]. As a typical feature of yeast iso–1 cyt c, the Lys72 of the protein is trimethylated [26,32].

In this manuscript, the methyl of the trimethylated Lys72 (Lys72me3) of cyt c was used as a natural probe for the conformational transition of cyt c. Methyl has been widely used as a sensitive NMR probe to observe protein structure due to its slow transversal relaxation [33,34,35]. The modified Lys72me3 has nine equivalent protons located on the surface of the protein, making it possible to be applied to monitor the conformational transition of natural isotopic abundance protein in a large complex. ^1^H–^14^N HSQC is powerful at simplifying spectra of trimethylated proteins [36]; however, the small ^2^J_1H, 14N_ coupling constant makes it difficult to be used to probe the macromolecule complex. Herein, to accurately trace the modified methyl, 2D four–quantum–filtered maximum–quantum correlation ^1^H–^13^C HMQC (MAXY–HMQC) was applied to filter out signals other than methyl groups. MAXY–HMQC, developed by Liu et al. [37], can selectively detect the signals of methyl, methylene, and methylene separately and simplify the spectra of biomolecules [38,39]. The chemical shift of the modified Lys72me3 methyl is far away from the side chain methyls both in the ^1^H and ^13^C dimensions; therefore, ^1^H–^13^C NMR can be used to trace the methyl. Because the trimethylated group has nine equivalent protons, the method is sensitive to the conformational changes of cyt c [32] and used to probe the conformational change of cyt c upon interaction with different contents of CL, even though the high concentration of CL and cyt c interact to form large complexes [23,24].

The results show that the conformation of cyt c changed from a native conformation to two extended conformations upon interaction with high content of CL, in which the Fe–Met80 bond was broken and resulted in a bis–His hexa–coordination and a penta–coordination heme simultaneously. The state with a penta–coordination heme seems to be more stable and preferable upon interaction with CL, as the content of this state gradually increased with further enhancement of the ratio of CL to cyt c. These results indicate that the preferred conformation of cyt c was different in the presence of different contents of CL, making it reasonable that cyt c obtains high peroxidase activity, as the heme is more exposed in the preferable penta–coordination state [40], which is consistent with the characterization of other peroxidases [41].

## 2. Materials and Methods

### 2.1. Chemicals

Yeast iso–1 cyt c and CL were purchased from Sigma Aldrich (Merck, Burlington, MA, USA). Cyt c was further purified using the method reported previously [42] and dissolved in phosphate buffer (PB, 20 mM, pH 7.0) (Sinopharm Chemical ReagentCo., Ltd., Shanghai, China) before use. The purity of the protein was then identified by mass spectrometry. The CL liposome was prepared according to a previously reported protocol [43,44]. A 5 mL ethanol solution of CL was evaporated under vacuum using a rotary evaporator at 318 K until the liquid was converted into an adherent film. Then, a 1 mL precooled solution of 20 mM PB was used to dissolve the CL film at 277 K. After that, the solution was sonicated in an ice bath for 15 min to obtain dispersed and uniform CL liposomes. The CL liposome was refrigerated (277 K) and sonicated for 10 min before use. The particle size and uniformity of the prepared CL liposome were measured by using dynamic light scattering (DynaPro NanoStar, Wyatt, Santa Barbara, CA, USA).

### 2.2. NMR Experiments

All NMR experiments were conducted in a Bruker Avance III 850 MHz NMR spectrometer equipped with a TXI cryoprobe. All the spectra were acquired at 298 K. To identify the modified methyl groups, 2D MAXY–HMQC was performed [38], in which the spectral widths of ^1^H and ^13^C dimensions were 15.00 ppm and 70.00 ppm, respectively.

To trace the change of the modified methyl groups more sensitively, 2D ^1^H–^13^C HSQC was used to replace the MAXY–HMQC spectra. For the HSQC spectra, the spectral widths of ^1^H and ^13^C dimensions were 15.00 ppm and 20.00 ppm, respectively. The data were acquired with complex sampling points of 2048 and 64 for the ^1^H and ^13^C dimensions, respectively. The number of scans was 64. The experimental data were processed using Bruker Topspin 4.0.1 software (Bruker, Billerica, MA, USA).

### 2.3. CD, Fluorescence, and –Visible Absorption Spectra Experiments

To further identify the possible characterization of the conformational transition, CD, fluorescence, and UV–visible absorption spectra were also obtained. CD experiments were carried out using a Chirascan CD spectrometer (Applied Photophysics, Leatherhead, UK) in the wavelength range of 190–260 nm with a 0.1 cm cuvette. Fluorescence and UV–visible absorption experiments were carried out using a SpectraMax i3x multimode plate reader (Molecular Devices, San Jose, CA, USA). Fluorescence emission spectra were recorded in the wavelength range of 300–500 nm (sensitive to the Trp50 to heme distance) with an excitation wavelength of 289 nm and in the wavelength range of 465–600 nm (thioflavin T fluorescent) with an excitation wavelength of 440 nm, respectively. The UV–visible absorption spectra were conducted in the wavelengths of 350–450 nm and 600–800 nm, respectively.

## 3. Results

### 3.1. Identification of Different Conformations of Cyt C at a Natural Isotopic Abundance

**Figure 1:**
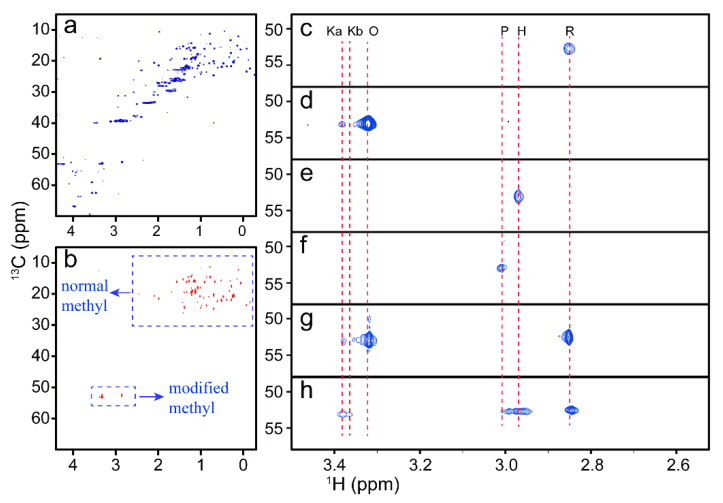
NMR spectra of cyt c with different conformations. (**a**,**b**) ^1^H–^13^C HSQC and four–quantum–filtered MAXY–HMQC spectra of cyt c, respectively. (**c**–**h**) Enlarged four–quantum–filtered ^1^H–^13^C MAXY–HMQC spectra of cyt c in different conditions. (**c**–**f**) Spectra of 0.3 mM cyt c in the presence of 1.0 mM SLA, 1.0 mM K_3_[Fe(CN)_6_], 1.0 M GuHCl, and 1.0 mM H_2_O_2_, respectively. (**g**,**h**) Spectra of 0.3 mM cyt c at pH 7.0 and 10.4, respectively.

To identify the resonance of Lys72me3 from different cyt c conformations, four–quantum–filtered ^1^H–^13^C MAXY–HMQC was applied to monitor the methyl of cyt c in different conditions. In the four–quantum–filtered MAXY–HMQC spectra, theoretically, except those of methyl groups, all signals from other groups are unobservable, as shown in Figure 1. Compared with the normal ^1^H–^13^C HSQC (Figure 1a), the signals in the four–quantum–filtered ^1^H–^13^C MAXY–HMQC spectra of cyt c are simplified (Figure 1b).

Because the ^13^C and ^1^H chemical shifts of the modified methyl groups are much larger than those of the other side chain, the signals of Lys72me3 can easily be identified by observing the signals in the low–field region of the spectra, as indicated in Figure 1b.

The enlarged low–field regions of the four–quantum–filtered ^1^H–^13^C MAXY–HMQC spectra of cyt c under different conditions are shown in Figure 1. The resonances of Lys72me3 in the native ferrous Fe–Met80 state (denoted R state) and in the ferric Fe–Met80 state (denoted O state) of cyt c can be assigned easily to the signals with ^1^H chemical shifts of 2.85 ppm and 3.31 ppm, respectively [45], according to the MAXY–HMQC spectra of cyt c with extra sodium L–ascorbate (SLA) (Figure 1c) and K_3_[Fe(CN)_6_] (Figure 1d). In Figure 1d, the weak signal at 3.38 ppm in the ^1^H dimension can be assigned to another ferric conformer with a different coordination form of heme [36]. There are two non–native states for cyt c, both with Fe–Lys coordination (denoted K state). They are states with Fe–Lys73 (denoted Ka state) and Fe–Lys79 (denoted Kb state) coordination, respectively. The two states predominate under acidic and alkaline conditions, respectively [46,47]. Therefore, we infer the weak signal comes from the Ka state because it also exists in the spectra under alkaline conditions (Figure 1h). The existence of this non–native conformer may come from the oxidative stress during protein extraction and purification because the state is found to exist when the cells undergo oxidative stress [16].

The spectra of the denatured protein vary by using different reagents (Figure 1e,f). The ^1^H chemical shift of the resonance in the spectra with extra GuHCl is lower than that with extra H_2_O_2_. This result seems to be rational, as it was reported previously that the conformers of cyt c denatured by GuHCl and H_2_O_2_ are structures with a ferric bis–His hexa–coordination (denoted H state) and penta–coordination heme (denoted P state), respectively [48,49]. Accordingly, the resonances with ^1^H chemical shifts of 2.98 and 3.01 ppm are partially unfolded cyt c in the H and P states, respectively [50,51]. It was reported that His26 was involved in Fe coordination only when His33 was missing [52]; therefore, this H state observed may mainly be a state with Fe–His33 coordination. There are three signals that coexist in the spectra of cyt c at pH 7.0 (Figure 1g), indicating that the O, R, and K states coexist under neutral conditions. This result coincides with the ^14^N–filtered NMR experiments reported previously [36]. Under alkaline conditions (Figure 1h), three different conformers coexist, as three signals were found in the MAXY–HMQC spectra of cyt c at pH 10.4. According to the assignments mentioned above, this result indicates that part of cyt c is still folded in the K state, whereas part of cyt c becomes partially unfolded in the H state.

The above results show that six different conformations of cyt c can be identified by measuring the modified methyl using NMR; therefore, it suggests that methyl–selective NMR detection is powerful to study the conformational transition of methylated protein at natural isotopic abundance.

### 3.2. Trace of Conformational Changes of Cyt C upon Interaction with CL

**Figure 2:**
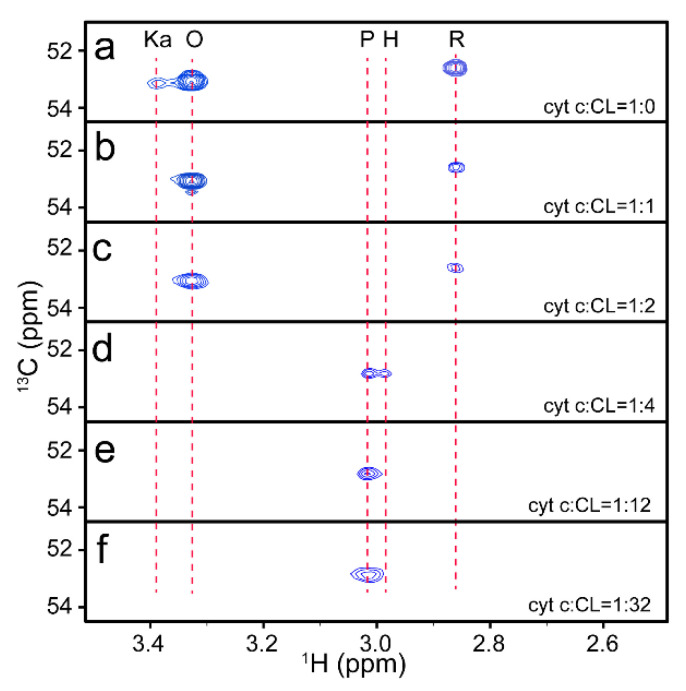
^1^H–^13^C HSQC spectra of 0.3 mM cyt c in different contents of CL liposome. (**a**) cyt c: CL = 1:0, (**b**) cyt c:CL = 1:1, (**c**) cyt c:CL = 1:2, (**d**) cyt c:CL = 1:4, (**e**) cyt c:CL = 1:12, (**f**) cyt c:CL = 1:32.

Lys72me3 is a sensitive probe for the conformational transition of cyt c; therefore, the interaction between cyt c and CL was monitored by observing the modified methyl using NMR spectroscopy. Because the resonance of K72me3 is distinguishable from other resonances, 2D ^1^H–^13^C HSQC spectra were applied to monitor the conformational transition of cyt c upon interaction with CL for higher sensitivity. The results are shown in Figure 2, in which the region that Lys72me3 locates is enlarged. Three resonances corresponding to cyt c in the R, O, and Ka states coexist in the absence of the CL liposome, which is the same as that of the MAXY–HMQC spectra, indicating that ^1^H–^13^C HSQC can be used to trace the change of Lys72me3.

With the addition of the CL liposome, the NMR spectra of cyt c changed by several degrees. At low CL/cyt c ratios, all three signals were gradually weakened (Figure 2b,c) and disappeared with increasing CL concentration from 1 to 4 times cyt c. This indicates that all conformers of cyt c interact with CL. Cyt c, when bound to large, slow–tumbling liposomes, has a higher correlation time, causing a decrease in NMR signal intensity in the HSQC spectra. Nevertheless, the relative content of the O state of cyt c increases in comparison with the other two conformers, which suggests that conformational transition and oxidization are induced by the interaction. The results coincide with the report that the addition of CL promotes the conversion of cyt c from ferrous state to ferric state [44].

It was found that the conformational transition continued to occur as the CL/cyt c ratios increased. When the CL content was 4 times that of cyt c, two new conformers appeared with the disappearance of the former three conformers. As assigned above, the two new conformers were cyt c in the H and P states, respectively. This result coincides with the report that the conformation of cyt c changed in combination with the CL liposome [18,19]. When the content of CL further increased, the resonance of P state increased with the disappearance of the resonance of H state.

Compared with the normal ^1^H–^15^N NMR detection, which cannot detect protein under high CL content [22], these results show that modified methyl detection is a powerful method to study the conformational transition of methylated protein and indicate that cyt c undergoes multiple conformational transformations upon interaction with different contents of CL. Upon interaction with CL, native O and R states of cyt c both become partially unfolded. However, unlike those reported before, two partially unfolded conformers of cyt c were found to coexist and transit to each other. When the content of CL further increased, the H–state protein transited into a P–state protein. It has been found that the content of CL in the outer membrane of the mitochondria usually increases along with apoptosis [4], and the exposure of heme is usually accompanied by higher peroxidase activity [51]. Therefore, these results indicate that cyt c preferred to be in a state with high peroxidase activity when inserted into the membrane of mitochondria. Because the increase of CL content induces the protein to be in a conformation with higher peroxidase activity, the migration of CL might also be an action against oxidative stress.

### 3.3. The Structure of Cyt C Becomes Expanded upon Interaction with CL

**Figure 3:**
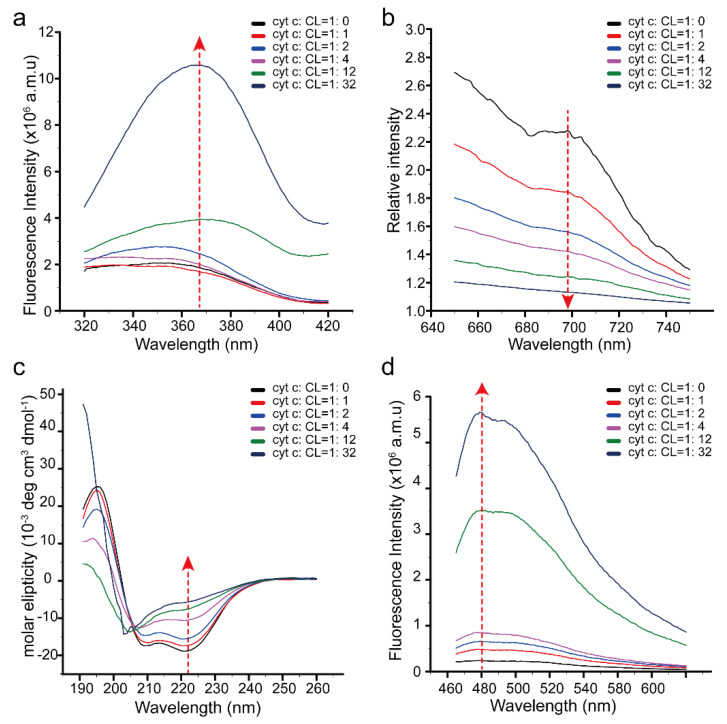
Conformational changes of cyt c with increasing CL. (**a**) Fluorescence spectrum of 0.02 mM cyt c in different contents of CL liposome. (**b**) UV–visible spectrum of 0.1 mM cyt c in different contents of CL liposome. (**c**) CD spectrum of 0.02 mM cyt c in different contents of CL liposome. (**d**) Fluorescence spectra of 0.02 mM cyt c mixed with ThT in different contents of CL liposome.

To further clarify the conformational change of cyt c upon interaction with CL in detail, fluorescence, UV–visible, and CD spectra were also obtained (Figure 3).

The intensity of the fluorescence representing the gradual exposure of Trp59 on cyt c enhanced with increasing CL content [53], which means that the binding of CL induces the exposure of the heme. A shift to a higher wavelength (red shift) was visible in the spectra by adding CL to the solution. This phenomenon is generally related to a shift from a less exposed to a more exposed Trp59, which also suggests the exposure of the heme. (Figure 3a). The decreased absorption at 695 nm in the UV–visible spectra indicates that the Fe–Met80 bond of cyt c is gradually broken [49] and suggests that CL binding causes the rupture of the Fe–Met80 bond of cyt c (Figure 3b). The CD spectrum indicates that the secondary structure of cyt c gradually changes [48], with a significant reduction in α–helix and an increase in β–sheets (Figure 3c). Extracting the secondary structure percentages from the CD spectra showed that the percentage of α–helix weakened from 59.6% to 24.9%, but the percentage of β–sheets strengthened from 8.2% to 24.6%. The increase in β–sheets has been further identified by using a thioflavin T (ThT) fluorescence probe, which specifically binds to β–sheets of protein [54]. The enhanced fluorescence of ThT at 480 nm indicated that the interaction induced the increase in β–sheets in cyt c (Figure 3d).

**Figure 4:**
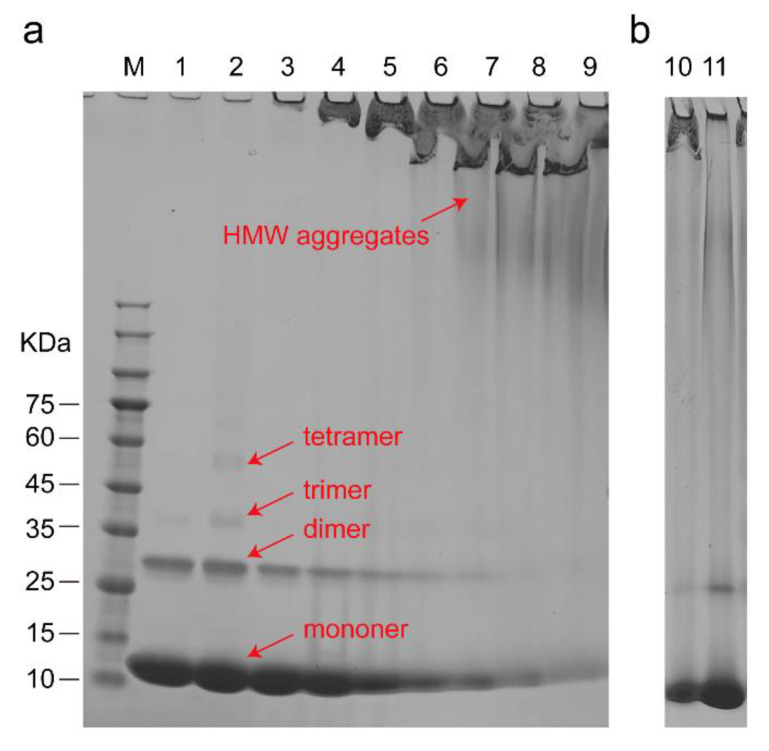
Effect of CL on the aggregation of cyt c. (**a**) SDS–PAGE of cyt c in the absence and presence of different contents of CL. M: unstained protein molecular weight marker; Lane 1: cyt c: CL = 1:0; Lane 2: cyt c: CL = 1:0.5; Lane 3 cyt c: CL = 1:1; Lane 4: cyt c: CL = 1:2; Lane 5: cyt c: CL = 1:4; Lane 6: cyt c: CL = 1:8; Lane 7: cyt c: CL = 1:12; Lane 8: cyt c: CL = 1:20; Lane 9: cyt c: CL = 1:32 and the influence of NaCl. (**b**) Effect of NaCl on the interaction of cyt c and CL; Lane 10: cyt c: CL = 1:32 with 200 mM NaCl; Lane 11: cyt c: CL = 1:32 with 500 mM NaCl.

Because the increase in β–sheets is usually associated with the aggregation of proteins [54], SDS–PAGE was used to monitor the aggregation states of cyt c (Figure 4a) and detect the possible driving force of the aggregation (Figure 4b). The result validates that the aggregation of cyt c can be induced by CL driven by electrostatic interaction and is consistent with recent research that the oligomerization of cyt c can be induced by CL [55]. The peroxidase activity of cyt c is increased by dimerization and oligomerization [56,57]. Therefore, the results indicate that CL induced the aggregation of cyt c, which also promoted the peroxidase activity of the protein. This aggregation may also be one of the main reasons for the decrease in and disappearance of NMR signals.

### 3.4. Redox Conversion of Cyt C

**Figure 5:**
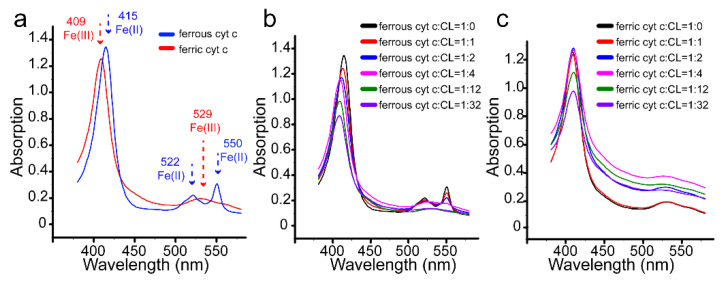
UV–visible spectra of 0.02 mM cyt c in the absence and presence of CL liposome. (**a**) Spectra of ferrous and ferric cyt c. (**b**) Spectra of ferrous cyt c in different contents of CL liposome. (**c**) Spectra ferric cyt c in different contents of CL liposome.

The redox states of cyt c are also important for the function of the protein [58]. The characteristic absorption in the UV–visible spectra of cyt c can be used to distinguish the ferric and ferrous states of the protein [49]. As shown in Figure 5a, the UV spectra of ferrous and ferric cyt c are different from each other. Ferrous and ferric cyt c are prepared by adding additional sodium ascorbate and K_3_[Fe(CN)_6_], respectively. In Figure 5b, for ferrous cyt c, the UV absorption at 522 nm and 550 nm gradually decreased with the increase in CL, the characterized absorption at 415 nm gradually blue–shifted to 409 nm, and a new absorption at 529 nm appeared. These new phenomena are typical characteristics of ferric cyt c. However, for ferric cyt c (Figure 5c), no blue or red shift appears in the UV spectra even at high content of CL liposome. This indicates that ferrous cyt c is oxidized to ferric cyt c, and the coordination of heme changes upon interaction with CL. This result is consistent with our observations by NMR and a previous report [44].

## 4. Discussion

The conformational changes of cyt c upon interaction with CL was found to play a key role in apoptosis; however, it is still a controversial issue, because the altered conformation of cyt c upon CL interaction is heterogeneously assembled during the exchange equilibrium [20] and sensitive to the conditions of the experiments.

To comprehensively reveal the conformational change of cyt c upon interaction with CL, and avoid unpredictable results that might come from protein labeling or mutations, the conformers of cyt c with natural isotopic abundance extracted from yeast upon interaction with CL were detected.

By using 2D four–quantum–filtered maximum–quantum correlation ^1^H–^13^C HMQC spectroscopy, six different conformations of cyt c with natural isotopic abundance were successfully identified, which demonstrates that this technique is superior in simplifying the NMR spectrum of methylated proteins. Therefore, it is a good tool to detect the conformational change of methylated proteins that are difficult to overexpress with the modification. As the modified groups usually have more equivalent protons, the method may also be used to study the protein under the abundance of natural isotopes.

**Figure 6:**
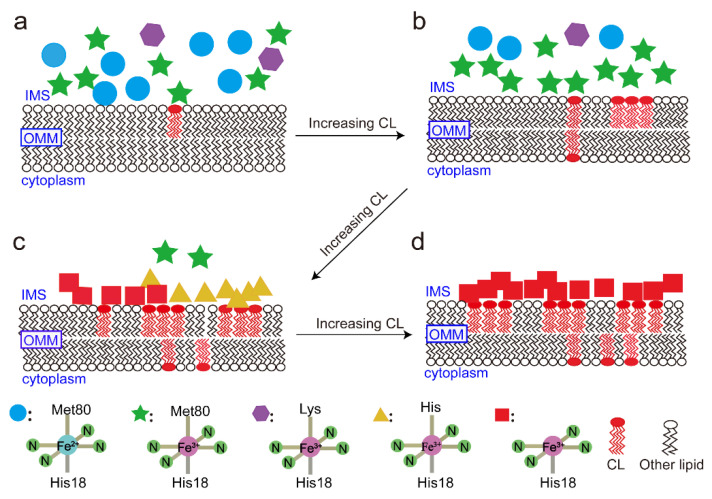
Model diagram of the interaction between cyt c and CL. (**a**) When the cell is in the normal condition, the distribution of CL in the OMM is limited, and there are interactions between CL and cyt c in different states. (**b**) When the CL is gradually transferred from the IMM to the OMM, the interaction between cyt c and CL induces the conformation transition of cyt c. (**c**) When the CL content continues to increase, the H state cyt c can transform into P state. (**d**) When the content of CL is further increased, the most of the cyt c are in P state.

By tracing the modified methyl of cyt c, it was found that the interaction between cyt c and CL leads to a series of conformational transitions of cyt c. Upon interaction, the structure of cyt c becomes expanded with the rupture of the normal Fe–Met80 bond and the exposure of heme. Because the content of CL in the OMM increases with apoptosis, the conformational transitions of cyt c may also be a stress reaction to apoptosis, as can be described in the diagram shown in Figure 6.

When the cell is in the normal condition, the distribution of CL in the OMM is limited, and interaction with CL makes cyt c in different normal states (O, R, and Ka states) all insert into the membrane. However, as the content of CL is relatively lower, only part of cyt c is inserted (Figure 6a).

When CL is gradually transferred from the IMM to the OMM stimulated by oxidative stress or apoptosis, the interaction between cyt c and CL in OMM enhances, which, in turn, induces the conformation transition of cyt c (Figure 6b,c). The Fe–Met80 bond in the native structure of cyt c is broken, and the protein appears to be partially unfolded with more β–sheets, some of which are in the H state, and some of which are in the P state. At the same time, the protein becomes aggregated. This aggregation, in turn, increases the insertion number of the protein in the membrane. Additionally, it was found that the conformation of cyt c in the H state can transit to the P state upon interaction with further increased content of CL (Figure 6d).

In summary, the structure of cyt c becomes partially unfolded with more exposed heme upon interactions with increased content of CL. Because Fe is the core of the protein for oxidation and reduction, it makes it reasonable that yeast cyt c has high power against apoptosis [59]. Additionally, cyt c is oxidized upon interaction with CL, which might correlate with the oxidation of CL, which, in turn, alters the permeability of the OMM of the mitochondria, and then causes the release of the protein into the cytoplasm, triggering apoptosis.
